# Recurrent *PDGFRB* mutations in unicentric Castleman disease

**DOI:** 10.1038/s41375-018-0323-6

**Published:** 2019-01-03

**Authors:** Zhaoming Li, Xuan Lan, Chaoping Li, Yanjie Zhang, Yingjun Wang, Weili Xue, Lisha Lu, Mengyuan Jin, Zhiyuan Zhou, Xinhua Wang, Ling Li, Lei Zhang, Xin Li, Xiaorui Fu, Zhenchang Sun, Jingjing Wu, Xudong Zhang, Hui Yu, Feifei Nan, Yu Chang, Jiaqin Yan, Xiaolong Wu, Guannan Wang, Dandan Zhang, Yuan Zhang, Ken H. Young, Mingzhi Zhang

**Affiliations:** 1 Department of Oncology, the First Affiliated Hospital of Zhengzhou University, Zhengzhou, 450052 China; 2 Lymphoma Diagnosis and Treatment Center of Henan Province, Zhengzhou, 450000 China; 3 Institute of Clinical Medicine, the First Affiliated Hospital of Zhengzhou University, Zhengzhou, 450052 China; 4 Department of Pathology, the First Affiliated Hospital of Zhengzhou University, Zhengzhou, 450052 China; 5 The Academy of Medical Science of Zhengzhou University, Zhengzhou, 450052 China; 6 Department of Hematopathology, the University of Texas MD Anderson Cancer Center, Houston, TX 77030 USA

**Keywords:** Cancer genomics, Haematological cancer

## To the Editor:

Castleman disease (CD) is a rare heterogeneous group of lymphoproliferative disorders that share common lymph node histopathological features [1, 2]. It can be classified into unicentric CD (UCD), HHV-8-positive multicentric CD (MCD) and HHV-8-negative MCD (also called idiopathic MCD or iMCD) according to the number of swollen lymph nodes, Kaposi sarcoma-associated herpesvirus/human herpesvirus-8 (HHV-8) infection status and clinical manifestations [2, 3]. UCD typically involves a slow-growing lymph node showing characteristic “Castleman-like” histopathologic changes, which is rarely life-threatening [4]. Conversely, both HHV-8–positive MCD and iMCD are characterized by enlarged lymph nodes in multiple regions, systemic inflammatory symptoms, and multiple organ dysfunction caused by dysregulation of cytokines [5].

HHV-8 has been established as the etiologic agent of HHV-8-associated MCD that most often occur in human immunodeficiency virus (HIV)-infected or otherwise immunocompromised individuals [6]. However, the pathogenesis of the other two subtypes of CD including UCD and iMCD is largely unknown, partly due to the rarity of the disorders. There are relatively few unbiased, genome-wide sequencing studies of CD, and those available have usually encompassed relatively small cohorts or gene panels [7–9]. Here, we carried out whole-exome sequencing in a cohort of 40 individuals with CD to illustrate the genetic landscape of this disease.

The study design is described in Supplementary Figure 1. In total, 75 CD patients (41 cases of UCD and 34 cases of iMCD) from the First Affiliated Hospital of Zhengzhou University and Tongji Hospital of Wuhan between 1 January 1998 and 31 December 2016 were included at the time of diagnosis. All cases were reviewed and interpreted independently by two experienced pathologists, and the diagnoses were made according to the generally accepted guidelines [10, 11]. We excluded patients with concomitant malignancies, HIV infection, and polyneuropathy, organomegaly, endocrinopathy, M-protein, and skin pigmentation syndrome as well as patients without sufficient clinical data. The study was conducted in accordance with the Declaration of Helsinki and with approval of the Institutional Review Board of the First Affiliated Hospital of Zhengzhou University. Written informed consent was obtained from all patients. Whole-exome sequencing was performed in 40 tumor and matched normal tissues from CD patients (discovery cohort, 18 cases of UCD, and 22 cases of iMCD), and then *PDGFRB* mutations encoding p.Asn666Ser were validated by targeted deep sequencing (>10,000×) in an independent cohort of 35 CD samples (validation cohort, 23 UCD, and 12 iMCD). Detailed description of exome and targeted deep sequencing analysis was provided in Supplemental methods.

To explore the genetic etiology of CD, we performed whole-exome sequencing in 40 patients with CD, including 18 cases of UCD, and 22 cases of iMCD (Supplementary Figure 1). The mean sequencing depth was 255×, and a mean of 98.6% of the target sequence was covered to a depth of at least 20× (Supplementary Table 1). A total of 1034 nonsilent mutations (median = 6, range: 0–284) were identified, including two cases of CD harboring more than 200 mutations each (Supplementary Table 2). The number of nonsilent mutations in CD was less than that reported in lymphomas [12]. It should be noted that the mutant allele frequencies were generally less than 0.25 (median = 0.11) in CD cases, which could have compromised sensitivity in detecting mutations (Supplementary Table 1).

**Fig. 1:**
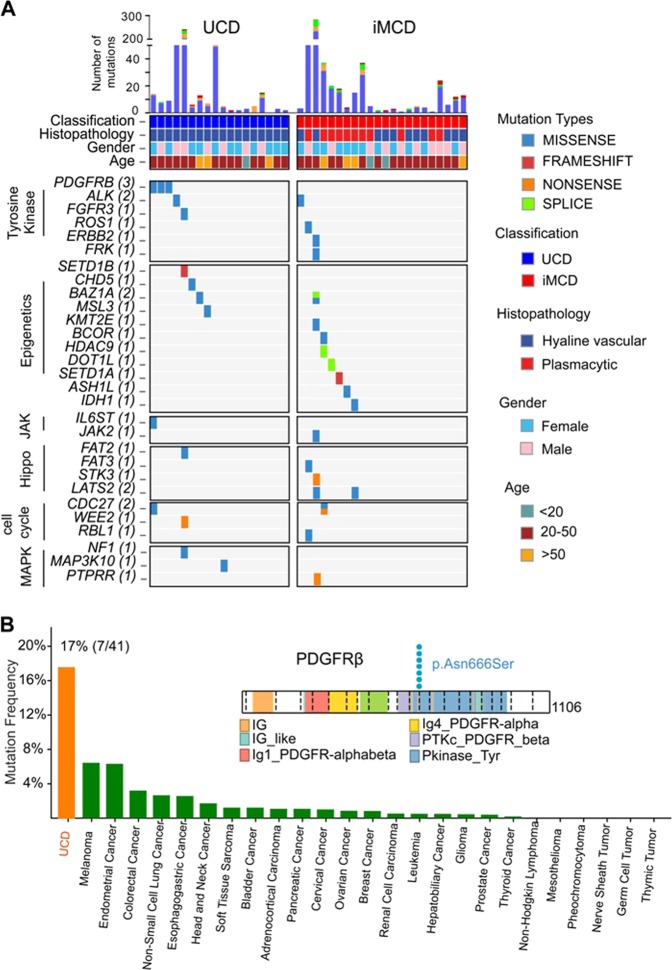
Whole-exome sequencing in 40 cases of CD. **a** Heat map of mutated genes in CD cases. These cases are separated into UCD set (*n* = 18) and iMCD set (*n* = 22). Each row represents a mutated gene in CD. Each column represents a patient sample. Blocks are color-coded by functional type of mutation. Top panel shows the number and type of nonsilent somatic mutations in each case. The mutations most likely to be related to CD pathogenesis are classified into the categories (indicated by different colors on the left): tyrosine kinase, epigenetic modifiers, Janus kinase/signal transducers and activators of transcription (JAK/STAT) pathway, Hippo pathway, cell cycle, and mitogen-activated protein kinase (MAPK) pathway. **b** Frequencies of *PDGFRB* mutations across cancer entities. Mutation frequencies obtained from http://www.cbioportal.org/public-portal/. UCD show the highest *PDGFRB* mutations frequency in cancer. Right panel shows the mapping of PDFGRB mutation sites in all of the UCD cases. Functional domains of the altered proteins are based on the UniProt database

The most frequently mutated gene was platelet-derived growth factor receptor b (*PDGFRB*), in which identical c.1997A > G mutations predicted to result in a p.Asn666Ser alteration were identified in 3 UCD samples (Fig. 1a). Prompted by this discovery, we screened *PDGFRB* mutations in 35 additional CD samples by targeted deep sequencing of the mutational hotspot (c.1997A > G; p.Asn666Ser) of *PDGFRB* (Supplementary Table 3). The validation cohort consists of 23 cases of UCD and 12 cases of iMCD. The three UCD cases harboring the *PDGFRB* Asn666Ser mutations from the discovery cohort were also verified by targeted deep sequencing (Supplementary Table 3). In total, *PDGFRB* mutations were found in 7 of the total 75 cases in the series (Fig. 1b and Supplementary Table 3). Notably, *PDGFRB* mutations only affected the patients with UCD, and all of them were hyaline-vascular variant (17%, 7 of 41). Comparison of the frequencies of *PDGFRB* mutations across different cancers revealed that UCD had the highest incidence of *PDGFRB* mutations across cancers (Fig. 1b). Moreover, no *PDGFRB* mutations encoding p.Asn666Ser were found in other hematologic neoplasms, suggesting that the *PDGFRB* mutations encoding p.Asn666Ser are highly specific to UCD. The relationship between the *PDGFRB* mutations and the clinical features of patients with UCD were further evaluated. However, no significant association was found between *PDGFRB* mutation status and clinical characteristics (Supplementary Table 4).

**Fig. 2:**
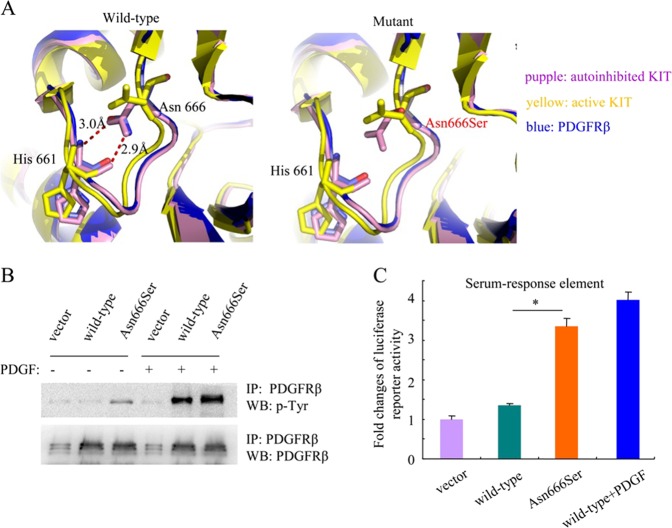
The PDGFRβ Asn666Ser mutant constitutively activated the receptor kinase activity. **a** A ribbon diagram shows the interaction between Asn666 and His661 in the PDGFRβ model (blue). The structure of the autoinhibited (purple) and active (yellow) forms of KIT kinase are shown for comparison. The p.Asn666Ser change would abolish the interaction linking Asn666 and His661. **b** Analysis of the phosphorylation and expression levels of the wild-type and mutated receptors by western blot. NIH3T3 cells stably expressing wild-type or mutant PDGFRβ were starved for 6 h and stimulated with PDGF-BB (20 ng/ml) for 15 min or left untreated before lysis. PDGFRβ was immunoprecipitated and analyzed by western blot experiments using an anti-phosphotyrosine antibody. **c** The activity of the PDGFRβ Asn666Ser mutant was analyzed in a luciferase reporter assay. NIH3T3 cells were transiently cotransfected with empty vector, wild-type or mutated *PDGFRB* receptors and a luciferase gene downstream of a SRE promoter. Four hours after transfection, cells were washed and treated or not with PDGF-BB (20 ng/ml) for 24 h. The histogram represents the fold changes in luciferase activity with SEM. Independent experiments were performed three times. *p < 0.05

The somatic mutations in PDFGRB encoding p.Asn666Ser identified herein have not been previously reported. To gain insight into its potential effects on receptor activity, we modeled the conformation of the cytoplasmic domain of PDGFRβ based on the crystallographic structures of human KIT kinase according to previous reports [13]. PDGFRβ and KIT are two related receptor tyrosine kinases showing similar structure. We compared the PDGFRβ model with the structures of the autoinhibited (PDB ID: 1T45) and active (PDB ID: 1PKG) forms of KIT. In the PDGFRβ model, the side chain of Asn666 participated in hydrogen-bonding interactions with the backbone of His661. A similar interaction was observed between Asn655 and His650 in the structure of the autoinhibited form of KIT kinase (Fig. 2a, left panel). However, in the active form of KIT kinase, the side chain of Asn655 was oriented in a different direction and no longer interacted with residue His650. The p.Asn666ser substitution in PDGFRβ would thus abolish the interaction between Asn666 and His661, altering the interactions in this area of the protein and possibly leading to a structure more similar to the active conformation of KIT kinase (Fig. 2a, right panel).

We next tested whether the Asn666Ser mutant constitutively activated the receptor kinase activity by western blot assay. As expected, the mutant showed robust ligand-independent autophosphorylation. In contrast, wild-type PDGFRβ was phosphorylated only in the presence of PDGF (Fig. 2b). These results were further confirmed by performing a luciferase reporter assay driven by a serum-response element (SRE), as readout of PDGFRβ signaling. In contrast to the unstimulated wild-type PDGFRβ, the Asn666Ser mutant constitutively activated SRE-dependent transcription, which was in line with the results revealed by western blot assay (Fig. 2c). These data suggest that the p.Asn666Ser substitution constitutively activates PDGFRβ.

We further analyzed the functional impact of the Asn666Ser mutant by testing its transforming potential. Focus formation assay was conducted in NIH3T3 fibroblasts transfected with wild-type or mutant PDGFRβ. As expected, the ability of cells transfected with Asn666Ser mutant to form foci was clearly enhanced compared with the cells transfected with wild-type PDGFRβ (Supplementary Figure 2A). In accordance with this observation, Asn666Ser mutant also conferred an IL-3-independent growth on the murine Ba/F3 cells, which is normally dependent on IL-3 for growth and survival (supplemental Fig. 2B). These data imply that Asn666Ser mutant has the potential to transform cells.

Until now, the cell type responsible for driving UCD pathogenesis has not been definitively identified. It has been hypothesized that UCD is most likely driven by a neoplastic stromal cell population [3, 4, 14, 15]. To determine the somatic origin of *PDGFRB* p.Asn666Ser mutations, we performed the BaseScope [16]—a novel mutation specific RNA in situ hybridization assay in archived formalin-fixed paraffin-embedded UCD tissue samples. Our results showed that BaseScope signals were exclusively detected in CD45− cells, which was enriched for nonhematopoietic stromal cells (Supplemental Figure 3). Therefore it was suggested that the *PDGFRB* p.Asn666Ser mutations were confined to non-hematopoietic stromal cells in UCD.

In this study, the recurrent *PDGFRB* mutations encoding p.Asn666Ser were detected in UCD patients, which strongly indicates that *PDGFRB* mutations in stromal cell might play a critical role in the pathogenesis of UCD.

## Supplementary information

Supplemental information

Figure S1

Figure S2

Figure S3

Supplemental tables
