# Supplementary material for: Recurrent PDGFRB mutations in unicentric Castleman disease
Source: Leukemia. 2019 Jan 3;33(4):1035–8. doi: 10.1038/s41375-018-0323-6 (PMC6484698; doi:10.1038/s41375-018-0323-6)
Supplement: Supplementary file 1 — Supplemental information [file 41375_2018_323_MOESM1_ESM.doc]

**Supplemental information**

**Methods**

**DNA extraction**

Genomic DNA from whole blood and FFPE samples was extracted using Blood & Cell Culture DNA Kit (Qiagen, Hilden, Germany) and QIAamp DNA FFPE Tissue Kit (Qiagen, Hilden, Germany), respectively. The quality and yield of purified DNA was assessed by fluorometry (Qubit, Invitrogen), Nanodrop 1000 spectrophotometer (Thermo Scientific, Wilmington, DE, USA) and gel electrophoresis.

**Whole-exome squencing and bioinformatic analysis**

Whole-exome squencing and bioinformatic analysis were performed by Beijing Genomics Institute (Beijing, China). Genomic DNA (1-1.5 µg) was fragmented by Covaris ultrasonicator into target peak size of 180-280 bp. The fragment ends were blunted and 5’phosphorylated with T4 polynucleotide kinase, T4 DNA polymerase, and Klenow Large Fragment (all from New England BioLabs). The 3’ ends are A-tailed using Klenow exo-minus (New England BioLabs), and fragments were ligated to Illumina paired-end adaptors. Ligation products were purified with Agencourt AMPure XP beads and enriched by PCR using Illumina PCR primers InPE1.0 and InPE2.0 and PCR primer indices. Pooled, indexed libraries were captured using the Agilent SureSelect Human All Exon, 50Mb kit (Agilent Technologies) according to the manufacturer’s protocol and sequenced on an Illumina HiSeq 2500 instrument. MuTect and GATK Somatic Indel Detector were used to identify somatic single nucleotide variants and short insertions and deletions, respectively. In order to further enhance the accuracy of somatic mutations, we removed sequencing artifacts or residual germline variations according to our own false positive mutation database, and then annotated the high-confidence variants with Oncotator[^3^](#_ENREF_3). Whole exome sequencing data have been deposited into the NCBI Sequence Read Archive, under Accession Code PRJNA482267.

**Targeted deep sequencing**

Genomic regions containing the mutational hotspot (c.1997A>G; p.Asn666Ser) of PDGFRB were amplified using the FastTarget^TM^ technology (Genesky Biotechnologies Inc, Shanghai, China). After multiple PCR reactions, DNA fragments were ligated with the adaptor by using Q5 DNA polymerase Kit (New England Biolabs, MA, USA), and further purified by Agencourt AMPure XP (Beckman Coulter, CA, USA). Next-generation sequencing of the amplification products was carried out by Illumina HiSeq 2500 sequencing platform (Illumina, Inc., San Diego, CA, USA), following the manufacturer’s standard protocols. The bioinformatic analysis was performed as described for the exome sequencing analysis. All candidate variants were manually inspected in IGV to exclude false positives.

**Cell culture**

NIH3T3, HEK293T and Ba/F3 cells were cultured in Dulbecco’s Modified Eagle’s Medium (DMEM, Gibco, Life Technologies) supplemented with 10% heat-inactivated FBS (Sigma-Aldrich, St Louis, MO, USA), 2 mM glutamine, 100 U/ml penicillin, and 100 µg/ml streptomycin (Invitrogen) in 5% CO_2_ atmosphere at 37 °C. Additionally, Ba/F3 cells were IL-3-dependent (500 U/ml). The identity of these cell lines was confirmed by DNA profiling (using short tandem repeats, STR), and they were tested negative for mycoplasma contamination using the Lonza MycoAlert Mycoplasma Detection Kit.

**Mutagenesis and constructs**

Full-length human *PDGFRB* cDNA was purchased from Sino Biological Inc. (Beijing, China). Mutagenesis for the creation of constructs encoding the p.Asn666Ser mutant was carried out using the Quick-Change Site-Directed Mutagenesis Kit (Agilent Technologies, Santa Clara, CA) according to the manufacturer’s instructions. These constructs encoding wild type and mutant *PDGFRB* were subcloned into the lentivirus-based expression vector pCDH-CMV-MCS-EF1-copGFP (System Biosciences, #CD511B-1) and pcDNA3.1 (Thermo Scientific, Waltham, MA, USA). All of the cDNA sequences were confirmed by Sanger sequencing.

**Viruses packing and cell infection**

For overexpression of wild type and mutant *PDGFRB*, each lentiviral vector was cotransfected with the psPAX2 packaging plasmid and the pMD2.G envelope plasmid into HEK293T cells using Lipofectamine 2000 (Invitrogen) according to the manufacturer’s protocol. Virus was harvested 72 hours post-transfection, and NIH3T3 or Ba/F3 cells were infected with these viruses in the presence of 8mg/mL polybrene. Infected cells expressing GFP were sorted on a flow cytometer (FACS Vantage SE, BD Bioscience, San Jose, CA, USA), and the purity of the sorted cell fractions consistently exceeded 90%.

**Immunoprecipitation**

The immunoprecipitation assay was performed using the Pierce™ Classic Magnetic IP/Co-IP kit (Thermo Scientific, Waltham, MA, USA) according to the manufacturer’s protocol. Briefly, cells (1.0 × 10^7^) were lysed in cold Pierce IP Lysis buffer and Halt™ Protease and Phosphatase Inhibitor cocktail (Thermo Scientific, Waltham, MA, USA) for 15 min on ice. Cell debris was removed by centrifugation at 13,000 × g for 10 min. Cell lysate (1000 µg) was combined with 10 μg of PDGFRβ (28E1) antibody and incubated overnight at 4ºC with rotation. The antigen sample/antibody mixture was added to the tube containing pre-washed magnetic beads (50 µl) and incubated at room temperature for 1 hour with mixing. The beads were collected with a magnetic stand, washed twice with IP Lysis/Wash buffer, and once with purified water, then the antigen/antibody complex was eluted.

**BaseScope assay**

BaseScope assays were performed according to guidelines form the supplier (Advanced Cell Diagnostics, Newark, CA). Sections were taken at 3μm thickness onto Superfrost plus slides (Fisher Scientific, Loughborough, UK) and allowed to dry overnight at room temperature (RT). Sections were then baked at 60 °C for 1 h, and then deparaffinizing in xylene (5 min, twice) and ethanol (2 min, twice), then drying by baking at 60 °C for 2 min. Pretreat 1 (hydrogen peroxide) was applied at RT for 10 min, Pretreat 2 (target retrieval) for 15 min at 100 ° C and Pretreat 3 (protease) for 30 min at 40 °C, with two rinses in distilled water between pretreatments. BaseScope probes were then applied for 2 h at 40 °C in a HybEZ oven before incubation with reagents AMP0 (30 min at 40 °C), AMP1 (15 min at 40 °C), AMP2 (30 min at 40 °C), AMP3 (30 min at 40 °C), AMP4 (15 min at 40 °C), AMP5 (30 min at RT) and AMP6 (15 min at RT). Slides were rinsed with wash buffer (2 × 2 min) between each AMP incubation. Finally slides were incubated with Fast Red for 10 min at room temperature in the dark. Slides were then used for sequential CD45 (Leica, Buffalo Grove, IL) immunohistochemistry staining as previously described[^4^](#_ENREF_4).

**Western blot analysis**

Cells were lysed in cold RIPA lysis buffer and a Halt™ Protease and Phosphatase Inhibitor Cocktail (Thermo Scientific, Waltham, MA, USA) for 20 min on ice. The cell lysates were clarified by centrifugation at 10000×g for 20 min. Proteins (10–25 μg) were resolved by SDS-PAGE and transferred onto nitrocellulose membranes (Amersham Biosciences, Piscataway, NJ, USA). The membranes were blocked in TBS-T buffer (20 mM Tris-HCl, pH 7.5, 150 mM NaCl and 0.05% Tween-20) containing 5% (w/v) non-fat milk at room temperature for 1 h and then probed at 4°C overnight with antibodies to detect PDGFRβ (28E1), phospho-Tyrosine (P-Tyr-100) from Cell Signaling Technology (Boston, MA, USA); Detection was carried out with the SuperSignal West Femto Maximum Sensitivity Substrate Trial Kit (Pierce, Rockford, IL, USA). The band images were digitally captured and quantified with a ChemiDoc™ XRS+ system (Bio-Rad Laboratories, Hercules, CA, USA).

**Luciferase assay**

NIH3T3 cells were seeded at 1.0×10^5^ cells/well in 12-well plates. Twenty-four hours later, cells were transiently cotransfected with the following constructs: the empty vector, wild-type or mutated PDGFRB receptors (500ng), the luciferase gene downstream of a serum response elements (SRE) promoter (200ng, firefly) and 20 ng of pRL-TK control vector (*Renilla*) by using Lipofectamine 2000 (Invitrogen, Life Technologies) as recommended by the manufacturers. Four hours after transfection, cells were washed and treated with or without PDGF-BB (20 ng/ml) for 24 hours. The luciferase activity was assessed using a GloMax instrument (Promega, Leiden, The Netherlands).

**Focus formation assay**

NIH3T3 cells were transduced with lentiviral supernatants expressing the empty vector, wild-type or mutated PDGFRB as described above. After sorting, cells were seeded in six-well plate and were maintained in DMEM with 10% heat-inactivated FBS in 5% CO_2_ atmosphere at 37 °C until foci formed. The cells were then fixed using methanol and stained with 0.25% crystal violet. The plates were dried and the focus density was photographed and quantified.

**Cytokine-independent growth assay in Ba/F3 cells**

Ba/F3 cells were maintained in medium supplemented with IL-3 (500 U/ml, PeproTech) and were transduced with lentiviral supernatants expressing the empty vector, wild-type or mutated PDGFRB receptors. After sorting, cells were cultured in medium supplemented with IL-3 for another 24 hours, and then washed three times and grew in the absence of IL-3. Cell growth and viability were monitored daily by Trypan blue using a TC10 automated cell counter (Bio-Rad Laboratories, Hercules, CA, USA). Independent experiment was performed three times.

**Structural modeling**

KIT and PDGFRβ are members of the same receptor tyrosine kinase family (class III) and possess a high degree of sequence identity in the cytoplasmic region. We modeled the conformation of the cytoplasmic domain of PDGFRβ based on the crystallographic structures of human KIT kinase according to previous reports. The PDGFRβ model was compared with the structures of the autoinhibited (PDB ID: 1T45) and active (PDB ID: 1PKG) forms of KIT by using the program PyMOL.

**Statistical analysis**

Independent experiments were performed at least three times. The average of different experiments was represented with standard error of the mean (SEM). Comparisons between and among groups were performed with Student’s t-test and analysis of variance (ANOVA), respectively. The association of PDGFRB mutations and clinicopathological characteristics was analyzed by chi-squared or Fisher’s two-tailed exact test. Statistical analysis was carried out using IBM SPSS Statistics 19 software (IBM Corp., Armonk, NY, USA). p<0.05 was considered significant.

**References**

1. do Valle IF, Giampieri E, Simonetti G, Padella A, Manfrini M, Ferrari A*, et al.* Optimized pipeline of MuTect and GATK tools to improve the detection of somatic single nucleotide polymorphisms in whole-exome sequencing data. *BMC bioinformatics* 2016 Nov 8; **17**(Suppl 12)**:** 341.

2. Cibulskis K, Lawrence MS, Carter SL, Sivachenko A, Jaffe D, Sougnez C*, et al.* Sensitive detection of somatic point mutations in impure and heterogeneous cancer samples. *Nature biotechnology* 2013 Mar; **31**(3)**:** 213-219.

3. Ramos AH, Lichtenstein L, Gupta M, Lawrence MS, Pugh TJ, Saksena G*, et al.* Oncotator: cancer variant annotation tool. *Human mutation* 2015 Apr; **36**(4)**:** E2423-2429.

4. Li Z, Lu L, Zhou Z, Xue W, Wang Y, Jin M*, et al.* Recurrent mutations in epigenetic modifiers and the PI3K/AKT/mTOR pathway in subcutaneous panniculitis-like T-cell lymphoma. *British journal of haematology* 2017 Mar 14.

**Supplementary Figure legends**

Supplementary Figure 1. The study design.

Supplementary Figure 2. (A) NIH3T3 cells transduced with empty vector, wild-type or mutated *PDGFRB* were seeded in six-well plate and were kept in medium until foci formed. Foci were then stained using crystal violet and counted. The average of three independent experiments is represented with SEM. *, p < 0.05. (B) Growth rate of Ba/F3 cells transduced with empty vector, wild-type or mutated *PDGFRB* receptors after removal of IL-3. Independent experiments were performed three times. *, p < 0.05.

Supplementary Figure 3. (A) Validation of BaseScope probes in HeLa cells transduced with wild-type or p.Asn666Ser mutant *PDGFRB.* (B) Representative images of the *PDGFRB* p.Asn666Ser probeset in archived formalin-fixed paraffin-embedded UCD tissue samples. Probe binding is visualized as punctate red dots (indicated by red arrow). Scale bars represent 50 micron.
