# Supplementary figures and images for: Recurrent PDGFRB mutations in unicentric Castleman disease
Source: Leukemia. 2019 Jan 3;33(4):1035–8. doi: 10.1038/s41375-018-0323-6 (PMC6484698; doi:10.1038/s41375-018-0323-6)

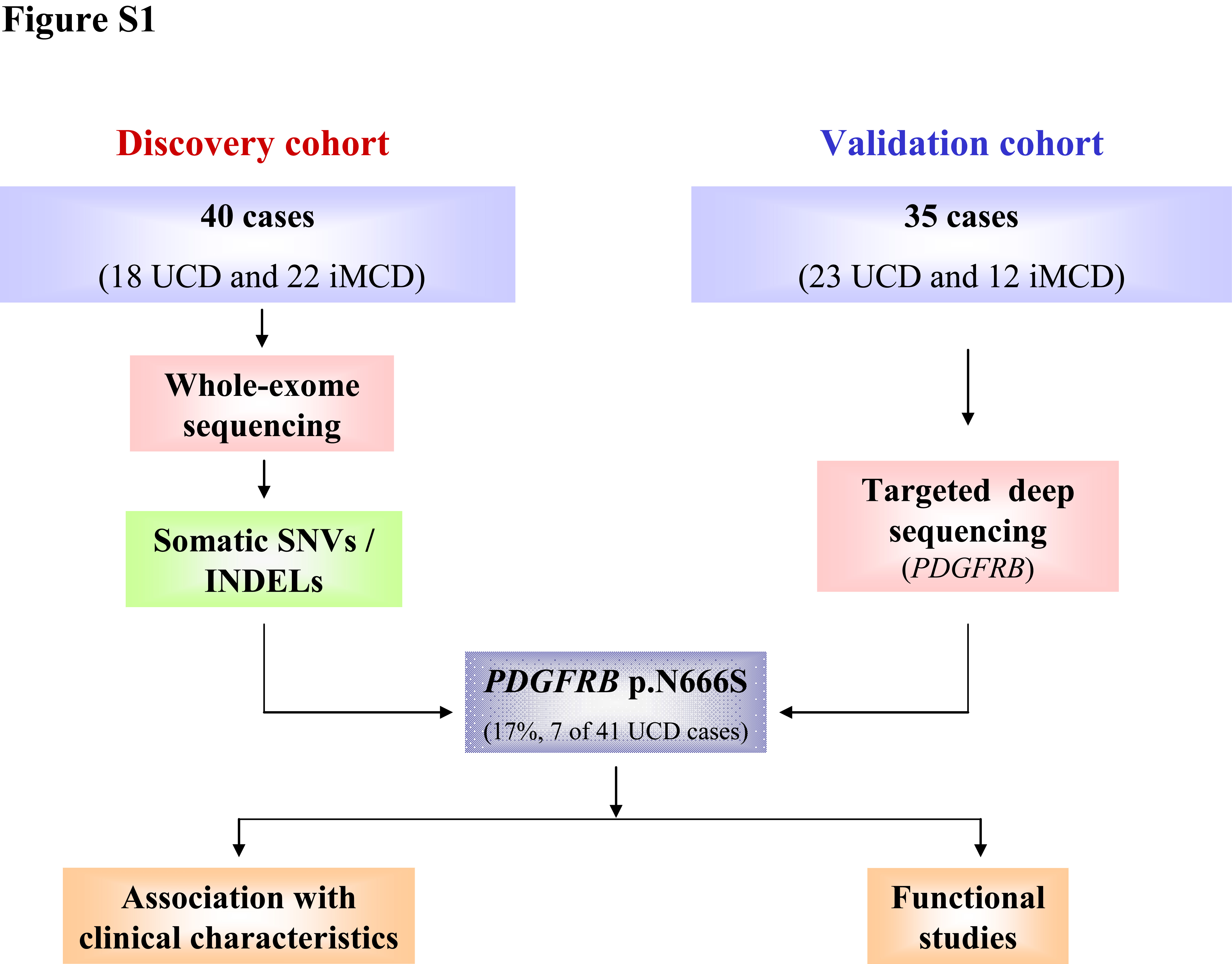

Supplement: Supplementary file 2 — Figure S1 [file 41375_2018_323_MOESM2_ESM.jpg]

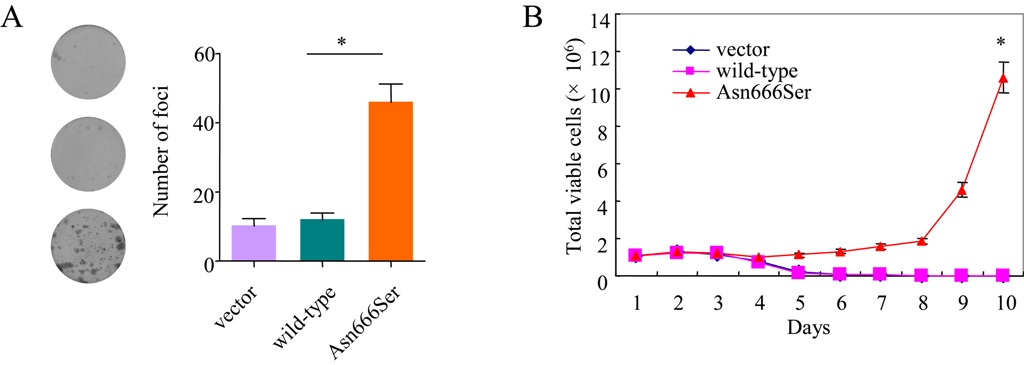

Supplement: Supplementary file 3 — Figure S2 [file 41375_2018_323_MOESM3_ESM.jpg]

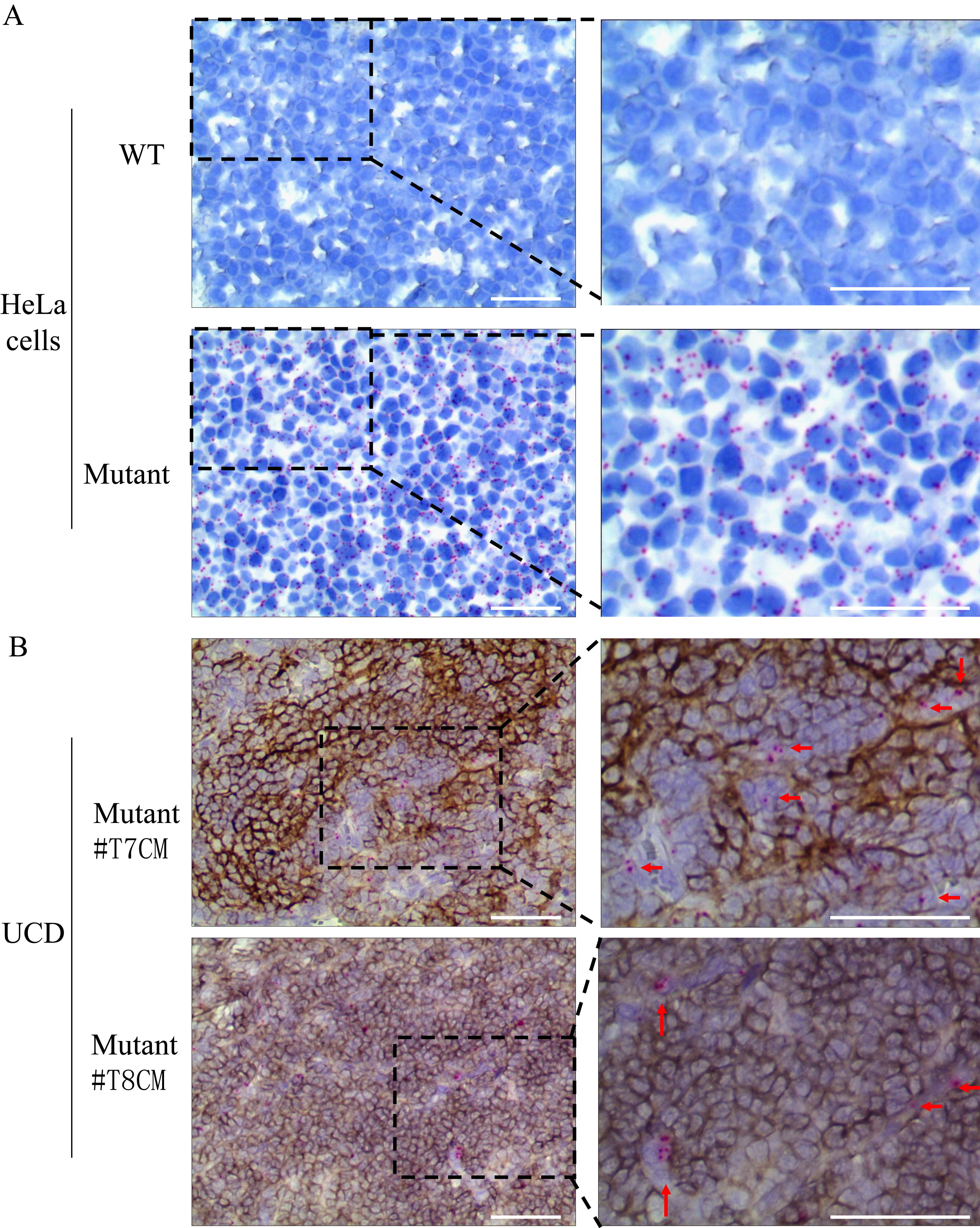

Supplement: Supplementary file 4 — Figure S3 [file 41375_2018_323_MOESM4_ESM.jpg]
